# Target of rapamycin signalling mediates the lifespan-extending effects of dietary restriction by essential amino acid alteration

**DOI:** 10.18632/aging.100665

**Published:** 2014-05-19

**Authors:** Sahar Emran, Mingyao Yang, Xiaoli He, Jelle Zandveld, Matthew D. W. Piper

**Affiliations:** ^1^Institute of Healthy Ageing, Department of Genetics, Evolution and Environment, University College London, London WC1E 6BT, United Kingdom; ^2^Institute of Animal Genetics and Breeding, Sichuan Agricultural University, Chengdu, Sichuan, 611130, China; ^3^Laboratory of Genetics, Wageningen University and Research Centre, 6708 PB, Wageningen, The Netherlands

**Keywords:** Drosophila melanogaster, rapamycin, target of rapamycin signalling, phenotyping, lifespan, stress response, essential amino acids

## Abstract

Dietary restriction (DR), defined as a moderate reduction in food intake short of malnutrition, has been shown to extend healthy lifespan in a diverse range of organisms, from yeast to primates. Reduced signalling through the insulin/IGF-like (IIS) and Target of Rapamycin (TOR) signalling pathways also extend lifespan. In *Drosophila melanogaster* the lifespan benefits of DR can be reproduced by modulating only the essential amino acids in yeast based food. Here, we show that pharmacological downregulation of TOR signalling, but not reduced IIS, modulates the lifespan response to DR by amino acid alteration. Of the physiological responses flies exhibit upon DR, only increased body fat and decreased heat stress resistance phenotypes correlated with longevity via reduced TOR signalling. These data indicate that lowered dietary amino acids promote longevity via TOR, not by enhanced resistance to molecular damage, but through modified physiological conditions that favour fat accumulation.

## INTRODUCTION

Dietary restriction (DR) is an intervention whereby a considerable reduction of food intake, just short of malnutrition, extends lifespan. This has been demonstrated to be effective in a wide range of evolutionarily diverse organisms, from yeast [1] to invertebrates [2]and mammals [3], and is considered one of the most robust environmental interventions to extend lifespan in laboratory organisms. Moreover, the longevity promoting effects of DR are accompanied by a range of health benefits. DR rodents had a delayed onset or a lesser severity of age-related diseases such as cancer, autoimmune diseases and motor dysfunction [4–6] and improved memory [7]. In *C. elegans*, DR was shown to reduce proteotoxicity [8]. DR rhesus monkeys were found to have improved triglyceride, cholesterol and fasting glucose profiles, and a reduced incidence of diabetes, cancer, cardiovascular disease and brain atrophy [9].

The molecular mechanisms underlying the physiological changes elicited by DR have yet to be elucidated, however, experimental data point towards nutrient signalling pathways as playing an important role. The evolutionarily conserved Target of Rapamycin Complex 1 (TORC1) pathway senses amino acid availability and signals to enhance translation via activation of S6 kinase-1 (S6K1) and inhibition of eIF4E binding protein-1(4E-BP1). TORC1 also regulates transcription and autophagy in response to a range of signals, including nutrient availability, cellular energy levels, and growth factors, in such a way that growth rates match resources [10]. Experimental validation of a role for TORC1 in determining lifespan has come from a range of laboratory organisms. Lifespan extension by inhibition of TORC1 pathway genes has been demonstrated in *S.cerevisiae*[11], *C.elegans* [12], *D. Melanogaster* [13] and in mice [14–19]. How TORC1 inhibition promotes longevity is unknown.

Another nutrient sensing pathway that is commonly associated with modified ageing is the insulin/insulin-like growth factor signalling (IIS) network. Mutations in components of the IIS pathway have extended lifespan in a host of model organisms [20]. Because the IIS pathway senses nutrients, considerable effort has been made to assess the role for IIS in modulating the longevity responses to DR. While IIS does not seem to be solely accountable for DR, some experimental data suggest overlapping mechanisms for IIS- and DR-mediated lifespan extension [21].

Recent work has shown that adjustments to the dietary amino acid balance can mimic the benefits to lifespan by DR in *D. melanogaster* [22]. Supplementing a DR diet with the ten essential amino acids (EAA) phenocopy the effects of full feeding (FF) on lifespan and fecundity, indicating that the beneficial effects of DR are a consequence of improved amino acid balance. Experimentally, the addition of EAAs to DR (DR+EAA) offers a sharper instrument with which to dissect the potential causes of lifespan change in response to nutritional balance than the FF condition, which is achieved by increasing the concentration of dietary yeast. Here we characterize physiological and metabolic parameters that define DR and fully fed flies with the aim of identifying candidate factors for causation of the lifespan response to DR.

## RESULTS

### TORC1 signalling but not IIS signalling is required for the effect of EAA on lifespan and fecundity

**Figure 1:**
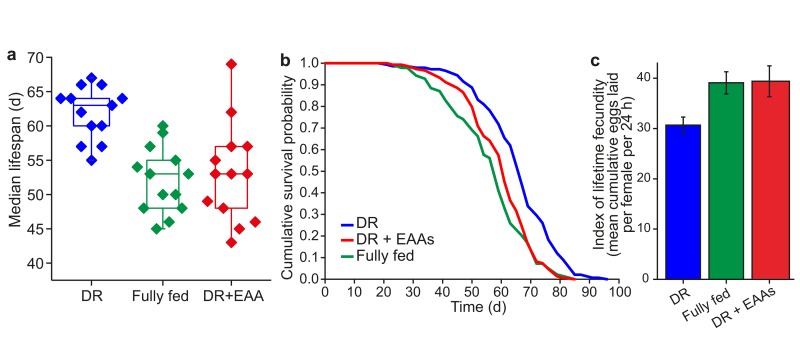
Amino acids mediate lifespan and fecundity changes under DR **(a)** Summary of *Drosophila* median lifespans under dietary restriction (DR), full feeding (FF) and essential amino acid supplementation of DR (DR+EAA) (*n*=13 biological replicates; DR vs FF, *P<*0.001; FF vs DR+EAA, *P=*0.9383; DR vs DR+EAA, *P=*0.002; Wilcoxon rank-sum test) **(b)** A representative lifespan experiment: adding EAAs to DR food shortened lifespan (*P*< 0.001) to that of FF flies (*P*=0.194); *n*=150 per treatment; compared using the log-rank test. **(c)** Adding EAAs to DR food increased egg-laying (*P*<0.001) to that of FF flies (*P<*0.936). Fecundity: mean±s.e.m.; *n*=15; compared using the Wilcoxon rank-sum test

Dietary restricted (DR) flies are longer-lived than fully fed flies, but produce fewer eggs. The effect of full feeding to shorten lifespan and increase egg laying can be mimicked by the addition of the 10 essential amino acids (EAA) to DR food (Figures 1a-1c).

**Figure 2:**
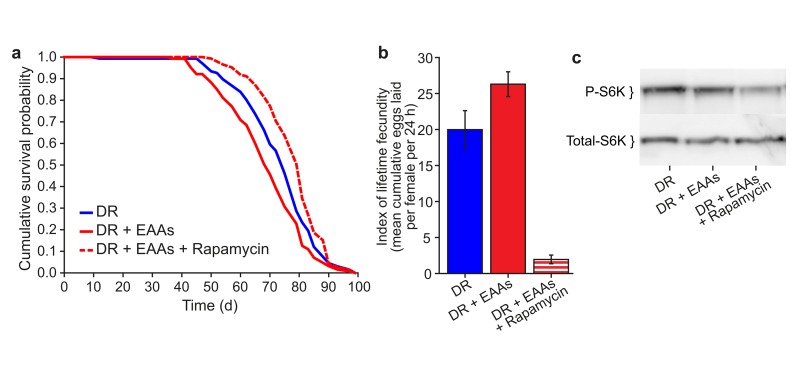
Effect of Rapamycin treatment on EAA-supplemented flies **(a)** Rapamycin treatment extended the lifespan of DR+EAA flies beyond that of DR (DR+EAA vs DR+EAA+Rapamycin, *P*<0.001; DR vs DR+EAA, *P*<0.012). *n*=150 per treatment; log-rank test. **(b)** Rapamycin treatment decreased the lifetime fecundity of DR+EAA flies (*P*<0.001). Fecundity: mean±s.e.m.; *n*=10; Wilcoxon rank-sum test. **(c)** Levels of phospho-T398-S6K were measured from whole-fly protein extracts. Treatment with rapamycin for 7 days decreased phospho-T398-S6K levels in DR+EAA+Rapamycin flies relative to DR+EAA flies

To assess the role of the longevity-associated nutrient signalling pathways as potential mediators of the effect of EAA on lifespan, we tested the response to DR of flies that are long lived due to deletion for genes encoding three of the*Drosophila* insulin-like peptides, (DILPs) *ilp*2, *ilp*3 and *ilp*5. We found no difference between the responses of wild type and DILP mutant flies to the addition of EAA to DR food, indicating that IIS is not required for the lifespan extension by DR (data not shown). In contrast, addition of the TORC1 inhibitor rapamycin extended the lifespan of flies on DR+EAA such that their lifespan was not shorter than those subjected to DR (Figure 2a). Rapamycin treatment also prevented the increase in egg laying seen for EAA addition to DR food, in fact egg laying was effectively blocked by rapamycin treatment. We also found that phosphorylation of the TORC1 target S6K was reduced by the addition of rapamycin (Figures 2b, 2c). Together, these data are consistent with TORC1 signalling playing a role in mediating the change in lifespan upon DR.

### EAA supplementation alters responses of DR flies to H_2_O_2_ stress, heat stress, starvation stress, and TAG levels

**Figure 3:**
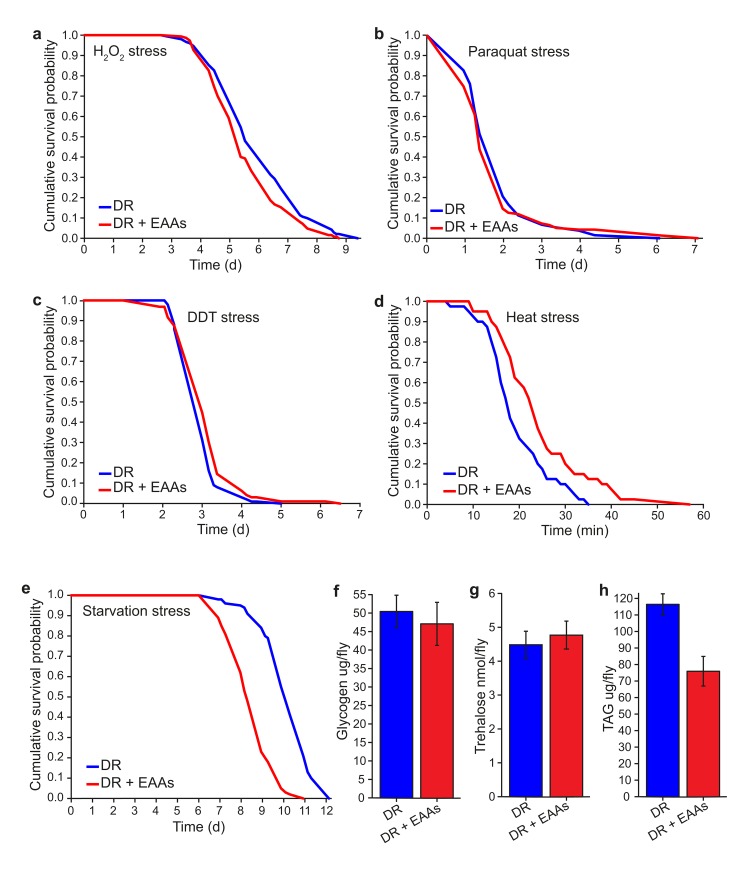
Phenotype comparisons between dietary restricted flies and those supplemented with EAAs **(a)** DR+EAA flies showed a decreased resistance to hydrogen peroxide toxicity compared to DR flies (*P*=0.013; *n*=150 flies per condition). **(b)** There was no difference between DR and DR+EAA flies in their sensitivity to paraquat stress (*P*=0.517; *n*=150 flies per condition). **(c)** DR+EAA flies showed only a marginal, but significantly improved tolerance to DDT compared to that of DR flies (*P*=0.042, *n*=100 flies per condition). **(d)** DR+EAA flies were significantly more resistant to a 39°C heat stress compared to DR flies (*P*<0.001; *n*=40 flies per condition). **(e)** DR+EAA flies were significantly more sensitive to starvation than DR flies (*P*<0.001; *n*=100 flies per condition). **(f)** After 7 days of treatment there was no difference in the amounts of glycogen measured for DR+EAA flies compared to DR flies (*P*=0.656; *n*= 6). **(g)** There was no difference in the levels of trehalose measured for DR+EAA flies compared to DR flies (*P*=0.630; *n*=6 flies per condition). **(h)** DR+EAA flies had significantly reduced levels of TAG compared to DR flies (*P*<0.001; *n*=6 flies per condition). For figures a-e, *P* values were calculated using the log-rank test. For figures f-h, *P* values were calculated by T-test, and error bars represent the s.e.m

We set out to identify phenotypic correlates of lifespan change under our dietary conditions in order to understand the causal mechanisms of increased lifespan under DR. Long-lived animal models often have an associated increase in the ability to resist environmental stresses and this is assumed to reflect a general increase in their health. Long-lived insulin/IGF-like signalling (IIS) mutant flies have been shown to be resistant to acute toxic doses of DDT, paraquat and hydrogen peroxide (H_2_O_2_) [23–25]. We tested whether long-lived DR flies are protected from the harmful effects of these compounds. We found that DR flies were significantly more resistant than DR+EAA flies to a toxic dose of H_2_O_2,_ whereas no difference was apparent for paraquat (Figures 3a, 3b). Surprisingly, DR flies were more sensitive to a toxic dose of DDT than DR+EAA flies (Figure 3c), indicating that, at least for DDT resistance, DR does not protect against this toxin in the same way that lowered insulin signalling does.

Long-lived DR *C. elegans* have increased resistance to heat stress [26,27]. Upon testing the response of flies to heat shock stress, we found that DR flies were significantly less resistant than DR+EAA flies (Figure 3d), indicating that longevity associated with amino acid reduction comes at a cost to heat stress resistance.

Finally, we found that DR flies showed greater resistance to starvation than DR+EAA flies (Figure 3e), suggesting a possible mechanistic relationship between longevity and starvation resistance. Resistance to starvation stress could depend on the availability of enhanced energy stores within the fly. While we found no difference between groups in the levels of the storage carbohydrates glycogen or trehalose (Figure 3f, 3g) we did find that DR flies had significantly higher levels of triacylglycerides (TAG) than DR+EAA flies (Figure 3h). It is possible that this difference in TAG levels is causative of the longevity differences between DR and DR+EAA flies such that increased TAG confers some benefit to survival.

### Increased TAG and decreased heat-stress resistance correlate with increased lifespan with DR

**Figure 4:**
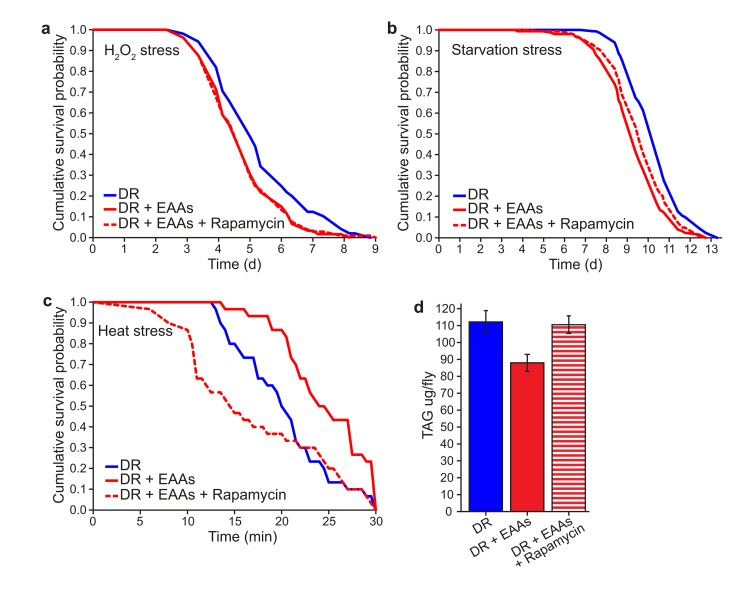
The effect of rapamycin to alter phenotypic differences between DR and DR+EAA flies **(a)** Rapamycin had no effect on the sensitivity of DR+EAA flies to H_2_O_2_ stress (*P*=0.963; *n*=105 flies per condition). **(b)** Rapamycin had no effect on the sensitivity of DR+EAA flies to starvation stress (*P*=0.071; *n*=150 flies per condition). **(c)** Rapamycin, like DR, increased the sensitivity of DR+EAA flies to a 39°C heat stress (*P*<0.001; *n*=30 flies per condition). For figures a-c, *P* values were calculated using the log-rank test. **(d)** Rapamycin treatment increased the triacyglyceride (TAG) levels of DR+EAA flies to the level of DR (*P*=0.011; *n*=6; T-test; error bars represent the s.e.m)

If the above phenotypes induced by DR are causally linked to longevity through reduced TORC1 signalling, it should be possible to reproduce the same physiological outcomes by treating flies with rapamycin. We therefore tested the effect of rapamycin on DR+EAA flies for H_2_O_2_ stress resistance, starvation sensitivity, heat shock stress resistance and TAG levels (Figures 4a-d). Of these, heat stress resistance and TAG levels changed upon rapamycin treatment of DR+EAA flies, such that the responses became more similar to DR flies; Like DR, rapamycin treatment increased the sensitivity of EAA-treated DR flies to a 39°C heat stress, and increased their TAG content. There was no effect of rapamycin on the response of EAA-treated flies to H_2_O_2_ stress or to starvation stress.

## DISCUSSION

We have described the physiological and metabolic features that define long-lived DR flies in order to understand the mechanisms by which longevity is achieved. Our data indicate that dietary amino acids modify TORC1 signalling, which in turn alters lifespan outcomes. We also found that both dietary amino acid manipulation and TORC1 modification in flies alter TAG levels, such that higher body fat may play a causal role in enhancing fly lifespan in response to dietary restriction.

We found that the lifespan of insulin-mutant flies responded in a similar way to DR as wild-types, indicating that reduced IIS is not required for the lifespan-extending effects of DR. This appears to contrast previous studies that have reported interacting effects of IIS on DR, such that lifespan modification in response to yeast dilution is abolished in some IIS mutants [22,23,28]. These differences could be due to the fact that in the current study we modulated lifespan by adjusting EAA alone, rather than yeast. In doing so, we report a markedly different sampling of nutritional space than for yeast dilution, since we change the ratio of EAAs to all other dietary components, such as lipids, carbohydrates, non-essential amino acids, vitamins and trace elements. This may also explain why the phenotypes of our long-lived flies are somewhat different from those of other organisms subjected to DR. Interestingly, our experiments also showed that long-lived DR flies had decreased resistance to DDT, which is the opposite phenotype seen for IIS mutant flies, in which longevity is accompanied by dFOXO-dependent DDT resistance [23,29]. Together, these data suggest that the beneficial effects on lifespan of DR can be achieved independently of IIS, similar to that reported by Tatar [21]. Moreover, it has been suggested that the effects of IIS on longevity are dependent on the status of TOR activity [30].

**Table 1:** Quantities of each of the essential amino acids added to 1l of 1xSYA food medium

Essential amino acid (Sigma-Aldrich)	Concentration in 1xSYA medium (g/l)
L-arginine	0.43
L-histidine	0.21
L-isoleucine	0.34
L-leucine	0.48
L-lysine	0.52
L-methionine	0.10
L-phenylalanine	0.26
L-threonine	0.37
L-tryptophan	0.09
L-valine	0.40

One of the strategies taken to understand the mechanisms by which dietary or genetic treatments enhance longevity is to seek out correlated physiological changes that may provide insights into the treatment's mode of action. A common mechanistic explanation for longevity requires enhancing systems to protect against the damaging side-effects of aerobic metabolism, such as that caused by oxidative stress or endogenous lipophilic toxins [31,32]. In our analyses, we found no evidence for broad-spectrum enhanced protection against stressors under DR. Thus, the mechanism for increased longevity under DR may not involve enhanced resistance to stress. Similar observations in studies on worms [33,34] has led to an alternative hypothesis that “hypertrophy” caused by inappropriate continuation of early-life growth programmes into later life is detrimental to an organism and causes ageing [35–38]. This explanation also implicates high levels of TOR signalling as its mechanism.

We found increased TAG levels correlated with longer life in our flies subjected to DR or rapamycin treatment. DR by yeast restriction in *Drosophila* has also been shown to increase lipid content [39–41], and several rodent studies show that higher fat levels correlate with increased lifespan [42-44]. In a recent study, Kapahi and colleagues showed that DR flies have increased TAG, and demonstrated an increased requirement for muscle-specific fatty-acid synthesis and breakdown in extending lifespan under DR [45]. Moreover, some long-lived TOR and IIS pathway mutants have increased fat levels [46–49]. Given that not all fat mutants are long-lived [50], it is likely that if fat levels are causally involved in extending life, the quality of fat accumulated is important. It would be interesting in future work to determine how lipid profiles change under different dietary conditions, to identify the specific types of lipids that are altered, and whether experimental manipulation can enhance lifespan.

## EXPERIMENTAL PROCEDURES

### General Methods

#### Standard laboratory food

Dietary restriction medium (1xSYA) contained100 g/l yeast (1x; MP Biomedicals, OH, USA), 50 g/l sucrose (Tate & Lyle, London, UK), 15 g/l agar (Sigma-Aldrich, Dorset, UK), and 30ml/l nipagin (Chemlink Specialities, Manchester, UK) and 3ml/l propionic acid (Sigma-Aldrich, Dorset, UK).This diet and its method of preparation is described in Bass et al., 2007 [51]. The fully fed medium (2xSYA) was prepared in the same way, except that it contained 200g/l yeast.

#### Experimental food

Rapamycin (LC Laboratories, MA, USA) was dissolved in ethanol and added to 1xSYA food at a final concentration of 200μM. Essential amino acids (Sigma-Aldrich, Dorset, UK) were dissolved in MiliQ water, and added to 1xSYA food at concentrations shown in Table 1. As control measures, ethanol alone was added to the food conditions that did not contain rapamycin, and water was added to food conditions that did not contain essential amino acids.

#### Fly stocks and husbandry

The wild-type Dahomey strain was originally collected in 1970 from Dahomey (now known as the Republic of Benin) and since maintained as a large outbred stock with overlapping generations at 25°C on a 12h light:12h dark cycle. These conditions allow for inter-generational breeding and the life expectancy of flies remain similar to that of newly caught wild flies [52]. Flies used for experimentation came from parental flies of the same age at egg laying, thereby controlling for the effects of parental age on lifespan [53].

Insulin-signalling mutant flies lacking the *Drosophila* insulin-like peptides (DILPs) *ilp*2, *ilp*3 and *ilp*5 were generated as described in Gronke et al., 2010 [23]. These flies were backcrossed into a control *white*^Dahomey^ background stock, which was derived by backcrossing *w*^1118^ into the outbred wild-type Dahomey background [24]. All mutations were back-crossed into their control backgrounds for a minimum of 6 generations.

#### Lifespan

All experiments were conducted at 25°C on a 12h light:12h dark cycle, at a constant humidity of 65%. Flies were reared at a standard larval density of ~300 flies per bottle, and all experimental adults were collected within a 12 hour period after eclosion. Flies were allowed to mate for 48 hours after eclosion before the experimental females were separated out under CO_2_ anaesthesia. Females were then randomly allocated to the experimental food treatments and housed in plastic vials containing food at a density of 10 flies per vial, with 15 vials per condition (*n*=150). Flies were transferred to a fresh food source 3 times per week, during which any deaths and censors were recorded.

#### Fecundity

Lifetime fecundity was measured as the cumulative total for days 7, 14, 21 and 28 of the mean number of eggs laid per female fly over each 24-hour period. Eggs in each vial were counted by eye using a light microscope after 18-24 hours exposure to flies.

#### Western blots

Protein extracts for western blot analysis were made from whole flies, sampled after 7 days of food treatment, using a TCA-based extraction protocol. 10μl of each sample was loaded into a 12% SDS-PAGE gel and blots were probed with anti-phospho-Thr398-S6K antibody (#9209, Cell Signaling Technologies, MA, USA), and total-S6K (re-made using a peptide sequence previously used to generate the total S6K antibody in Stewart et al., 1996 [54]). Both antibodies were used at a dilution of 1:12000 and normalised by probing with an anti-actin antibody at a dilution of 1:5000. Secondary antibodies conjugated to HRP (AbCam, Cambridge, UK) were used at a dilution of 1:5000, and the signals were detected by chemiluminescence.

### Stress Experiments

Experimental flies were reared and housed as described for the lifespan experiment. Mated female flies were kept on the experimental food types for 7 days before being transferred to the stress conditions.

#### Paraquat, DDT and H_2_O_2_ Stress

The orally administered stressors were as made up follows: 1xSYA containing 20mM paraquat (Sigma-Aldrich, Dorset, UK), 1xSYA containing 0.03% w/v DDT (Supelco Sigma-Aldrich, Dorset, UK), 1.5% agar medium containing 5% H_2_O_2_(Sigma-Aldrich, Dorset, UK) and 50g/l sucrose, or plain 1.5% agar medium for the starvation experiment.

#### Heat Shock

Experimental flies were transferred singly into dry empty 2ml glass vials, plugged with cotton wool and placed into a water bath set at 39°C. The time taken for each fly to fall onto its back and stop twitching (knockout) was recorded.

### Metabolic measurements

Experimental flies were reared and housed as described for the lifespan experiment. Mated female flies were kept on the experimental food types for 7 days before being frozen in liquid nitrogen. 6 replicas of 5 flies per condition were used for all metabolic measurements.

#### Triacylglyceride measurement

Flies per condition were homogenised in 0.05% Tween 20 (Sigma-Aldrich, Dorset, UK) according to Gronke et al., 2003 [55]. TAG content was quantified using the Triglyceride Infinity Reagent (Thermo Fisher Scientific, Surrey, UK).

#### Glycogen measurement

Flies were homogenised in 200μl saturated Na_2_SO_4_ solution and centrifuged for 1 min. 80μl of each sample was transferred to new Eppendorf tubes and 800μl chloroform:methanol (1:1) solution was added. Samples were centrifuged for 5 minutes and the supernatant was removed. The remaining pellet, containing precipitated glycogen, was resuspended in 1ml anthrone solution (anthrone in 50 ml 70% H_2_SO_4_) and incubated at 90°C for 20 minutes. 200μl of each sample was dispensed into the wells of a flat-bottomed 96-well plate, and the absorbance in each well was measured at 620nm and compared against a set of glycogen standards ranging from 0-2μg/μl (protocol adapted from Van Handel, 1965 [56]).

#### Trehalose measurement

Trehalose levels were measured using the Glucose Infinity Reagent (Thermo Fisher Scientific, Surrey, UK), as described in Broughton et al., 2005 [24].

## References

[R1] Jiang J, Jaruga E, Repnevskaya M, Jazwinski S (2000). An intervention resembling caloric restriction prolongs life span and retards aging in yeast. The FASEB Journal.

[R2] Klass M (1977). Aging in the nematode Caenorhabditis elegans: major biological and environmental factors influencing life span. Mechanisms of ageing and development.

[R3] McCay C (1935). The effect of retarded growth upon the length of life span and upon the ultimate body size. J Nutr.

[R4] Blackwell BN, Bucci TJ, Hart RW, Turturro A (1995). Longevity, Body Weight, and Neoplasia in Ad Libitum-Fed and Diet-Restricted C57BL6 Mice Fed NIH-31 Open Formula Diet. Toxicologic Pathology.

[R5] Weindruch RH, Kristie JA, Cheney KE, Walford RL (1979). Influence of controlled dietary restriction on immunologic function and aging. Federation Proceedings.

[R6] Ingram DK, Weindruch R, Spangler EL, Freeman JR, Walford RL (1987). Dietary restriction benefits learning and motor performance of aged mice. J Gerontol.

[R7] Stewart J, Mitchell J, Kalant N (1989). The effects of life-long food restriction on spatial memory in young and aged Fischer 344 rats measured in the eight-arm radial and the Morris water mazes. Neurobiology of aging.

[R8] Steinkraus K, Smith ED, Davis C, Carr D, Pendergrass WR, Sutphin GL, Kennedy BK, Kaeberlein M (2008). Dietary restriction suppresses proteotoxicity and enhances longevity by an hsf-1-dependent mechanism in Caenorhabditis elegans. Aging Cell.

[R9] Colman RJ, Anderson RM, Johnson SC, Kastman EK, Kosmatka KJ, Beasley TM, Allison DB, Cruzen C, Simmons HA, Kemnitz JW, Weindruch R (2009). Caloric restriction delays disease onset and mortality in rhesus monkeys. Science.

[R10] Martin DE, Hall MN (2005). The expanding TOR signaling network. Current Opinion in Cell Biology.

[R11] Kaeberlein M, Powers RW, Steffen KK, Westman EA, Hu D, Dang N, Kerr EO, Kirkland KT, Fields S, Kennedy BK (2005). Regulation of yeast replicative life span by TOR and Sch9 in response to nutrients. Science.

[R12] Vellai T, Takacs-Vellai K, Zhang Y, Kovacs AL, Orosz L, Müller F (2003). Genetics: influence of TOR kinase on lifespan in C. elegans. Nature.

[R13] Kapahi P, Zid BM, Harper T, Koslover D, Sapin V, Benzer S (2004). Regulation of Lifespan in Drosophila by Modulation of Genes in the TOR Signaling Pathway. Curr Biol.

[R14] Harrison DE, Strong R, Sharp ZD, Nelson JF, Astle CM, Flurkey K, Nadon NL, Wilkinson JE, Frenkel K, Carter CS, Pahor M, Javors MA, Fernandez E (2010). Rapamycin fed late in life extends lifespan in genetically heterogeneous mice. Nature.

[R15] Khapre RV, Kondratova AA, Patel S, Dubrovsky Y, Wrobel M, Antoch MP, Kondratov RV (2014). BMAL1-dependent regulation of the mTOR signaling pathway delays aging. Aging.

[R16] Johnson SC, Yanos ME, Kayser E-B, Quintana A, Sangesland M, Castanza A, Uhde L, Hui J, Wall VZ, Gagnidze A, Oh K, Wasko BM, Ramos FJ (2013). mTOR inhibition alleviates mitochondrial disease in a mouse model of Leigh syndrome. Science.

[R17] Ye L, Widlund AL, Sims CA, Lamming DW, Guan Y, Davis JG, Sabatini DM, Harrison DE, Vang O, Baur JA (2013). Rapamycin doses sufficient to extend lifespan do not compromise muscle mitochondrial content or endurance. Aging.

[R18] Komarova E, Antoch M, Novototskaya L, Chernova O, Paszkiewicz G, Leontieva O, Blagosklonny MV, Gudkov A (2014). Rapamycin extends lifespan and delays tumorigenesis in heterozygous p53+/- mice. Aging.

[R19] Popovich I, Anisimov V, Zabezhinski M, Semenchenko A, Tyndyk M, Yurova M, Blagosklonny MV (2014). Lifespan extension and cancer prevention in HER-2/neu transgenic mice treated with low intermittent doses of rapamycin. Cancer Biology & Therapy.

[R20] Fontana L, Partridge L, Longo VD (2010). Extending healthy life span--from yeast to humans. Science.

[R21] Min K-J, Yamamoto R, Buch S, Pankratz M, Tatar M (2008). Drosophila lifespan control by dietary restriction independent of insulin-like signaling. Aging Cell.

[R22] Grandison RC, Piper MDW, Partridge L (2009). Amino-acid imbalance explains extension of lifespan by dietary restriction in Drosophila. Nature.

[R23] Grönke S, Clarke D-F, Broughton S, Andrews TD, Partridge L (2010). Molecular evolution and functional characterization of Drosophila insulin-like peptides. PLoS Genetics.

[R24] Broughton SJ, Piper MDW, Ikeya T, Bass TM, Jacobson J, Driege Y, Martinez P, Hafen E, Withers DJ, Leevers SJ, Partridge L (2005). Longer lifespan, altered metabolism, and stress resistance in Drosophila from ablation of cells making insulin-like ligands. Proc Natl Acad Sci USA.

[R25] Slack C, Werz C, Wieser D, Alic N, Foley A, Stocker H, Withers DJ, Thornton JM, Hafen E, Partridge L (2010). Regulation of lifespan, metabolism, and stress responses by the Drosophila SH2B protein, Lnk. PLoS Genetics.

[R26] Chen D, Thomas EL, Kapahi P (2009). HIF-1 modulates dietary restriction-mediated lifespan extension via IRE-1 in Caenorhabditis elegans. PLoS Genetics.

[R27] Kaeberlein TL, Smith ED, Tsuchiya M, Welton KL, Thomas JH, Fields S, Kennedy BK, Kaeberlein M (2006). Lifespan extension in Caenorhabditis elegans by complete removal of food. Aging Cell.

[R28] Broughton SJ, Slack C, Alic N, Metaxakis A, Bass TM, Driege Y, Partridge L (2010). DILP-producing median neurosecretory cells in the Drosophila brain mediate the response of lifespan to nutrition. Aging Cell.

[R29] Slack C, Giannakou ME, Foley A, Goss M, Partridge L (2011). dFOXO-independent effects of reduced insulin-like signaling in Drosophila. Aging Cell.

[R30] Blagosklonny MV (2012). Once again on rapamycin-induced insulin resistance and longevity: despite of or owing to. Aging.

[R31] McElwee JJ, Schuster E, Blanc E, Thomas JH, Gems D (2004). Shared transcriptional signature in Caenorhabditis elegans Dauer larvae and long-lived daf-2 mutants implicates detoxification system in longevity assurance. The Journal of Biological Chemistry.

[R32] McElwee JJ, Schuster E, Blanc E, Piper MDW, Thomas JH, Patel DS, Selman C, Withers DJ, Thornton JM, Partridge L, Gems D (2007). Evolutionary conservation of regulated longevity assurance mechanisms. Genome Biology.

[R33] Gems D, Doonan R (2009). Antioxidant defense and aging in C. elegans. Cell Cycle.

[R34] Doonan R, McElwee JJ, Matthijssens F, Walker GA, Houthoofd K, Back P, Matscheski A, Vanfleteren JR, Gems D (2008). Against the oxidative damage theory of aging: superoxide dismutases protect against oxidative stress but have little or no effect on life span in Caenorhabditis elegans. Genes & Development.

[R35] Blagosklonny MV (2008). Ageing ROS or TOR. Cell Cycle.

[R36] Blagosklonny MV (2006). Cell senescence: hypertrophic arrest beyond the restriction point. Journal of Cellular Physiology.

[R37] Gems D, de la Guardia Y (2013). Alternative Perspectives on Aging in Caenorhabditis elegans: Reactive Oxygen Species or Hyperfunction?. Antioxidants & Redox Signaling.

[R38] Blagosklonny MV (2006). Aging and Immortality: Quasi-programmed senescence and its pharmacologic inhibition. Cell Cycle.

[R39] Skorupa DA, Dervisefendic A, Zwiener J, Pletcher SD (2008). Dietary composition specifies consumption, obesity, and lifespan in Drosophila melanogaster. Aging Cell.

[R40] Chippindale AK, Leroi M, Kim SB, Rose MR (1993). Phenotypic plasticity and selection in Drosophila life-history evolution. I. Nutrition and the cost of reproduction. J Evol Biol.

[R41] Bradley TJ, Simmons FH (1997). An analysis of resource allocation in response to dietary yeast in Drosophila melanogaster. Journal of Insect Physiology.

[R42] Liao C-Y, Rikke BA, Johnson TE, Gelfond JAL, Diaz V, Nelson JF (2011). Fat maintenance is a predictor of the murine lifespan response to dietary restriction. Aging Cell.

[R43] Harrison DE, Archer JR, Astle CM (1984). Effects of food restriction on aging: separation of food intake and adiposity. Proc Natl Acad Sci USA.

[R44] Miller RA, Buehner G, Chang Y, Harper JM, Sigler R, Smith-Wheelock M (2005). Methionine-deficient diet extends mouse lifespan, slows immune and lens aging, alters glucose, T4, IGF-I and insulin levels, and increases hepatocyte MIF levels and stress resistance. Aging Cell.

[R45] Katewa SD, Demontis F, Kolipinski M, Hubbard A, Gill MS, Perrimon N, Melov S, Kapahi P (2012). Intramyocellular Fatty-Acid Metabolism Plays a Critical Role in Mediating Responses to Dietary Restriction in Drosophila melanogaster. Cell Metabolism.

[R46] Böhni R, Riesgo-Escovar J, Oldham S, Brogiolo W, Stocker H, Andruss BF, Beckingham K, Hafen E (1999). Autonomous control of cell and organ size by CHICO, a Drosophila homolog of vertebrate IRS1-4. Cell.

[R47] Zhang H, Stallock JP, Ng JC, Reinhard C, Neufeld TP (2000). Regulation of cellular growth by the Drosophila target of rapamycin dTOR. Genes & Development.

[R48] Bjedov I, Toivonen JM, Kerr F, Slack C, Jacobson J, Foley A, Partridge L (2010). Mechanisms of life span extension by rapamycin in the fruit fly Drosophila melanogaster. Cell Metabolism.

[R49] Teleman AA, Chen Y, Cohen SM (2005). 4E-BP functions as a metabolic brake used under stress conditions but not during normal growth. Genes & Development.

[R50] Grönke S, Mildner A, Fellert S, Tennagels N, Petry S, Müller G, Jäckle H, Kühnlein RP (2005). Brummer lipase is an evolutionary conserved fat storage regulator in Drosophila. Cell Metabolism.

[R51] Bass TM, Grandison RC, Wong R, Martinez P, Partridge L, Piper MDW (2007). Optimization of dietary restriction protocols in Drosophila. J Gerontol A Biol Sci Med Sci.

[R52] Sgrò CM, Partridge L (2001). Laboratory adaptation of life history in Drosophila. The American Naturalist.

[R53] Priest N, Mackowiak B, Promislow D (2002). The role of parental age effects on the evolution of aging. Evolution.

[R54] Stewart MJ, Berry CO, Zilberman F, Thomas G, Kozma SC (1996). The Drosophila p70s6k homolog exhibits conserved regulatory elements and rapamycin sensitivity. Proc Natl Acad Sci USA.

[R55] Grönke S, Beller M, Fellert S, Ramakrishnan H, Jäckle H, Kühnlein RP (2003). Control of fat storage by a Drosophila PAT domain protein. Current Biology.

[R56] Van Handel E (1965). Microseparation of Glycogen, Sugars, and Lipids. Analytical Biochemistry.

